# Genome-Based Assessment of Antimicrobial Resistance and Virulence Potential of Isolates of Non-Pullorum/Gallinarum Salmonella Serovars Recovered from Dead Poultry in China

**DOI:** 10.1128/spectrum.00965-22

**Published:** 2022-06-21

**Authors:** Yan Li, Xiamei Kang, Abdelaziz Ed-Dra, Xiao Zhou, Chenghao Jia, Anja Müller, Yuqing Liu, Corinna Kehrenberg, Min Yue

**Affiliations:** a Department of Veterinary Medicine, Zhejiang University College of Animal Sciences, Hangzhou, China; b Hainan Institute of Zhejiang University, Sanya, China; c Zhejiang Provincial Key Laboratory of Preventive Veterinary Medicine, Hangzhou, China; d Institute for Veterinary Food Science, Faculty of Veterinary Medicine, Justus Liebig University Giessen, Giessen, Germany; e Shandong Key Laboratory of Animal Disease Control and Breeding, Institute of Animal Science and Veterinary Medicine, Shandong Academy of Agricultural Sciences, Jinan, China; f State Key Laboratory for Diagnosis and Treatment of Infectious Diseases, National Clinical Research Center for Infectious Diseases, National Medical Center for Infectious Diseases, The First Affiliated Hospital, College of Medicine, Zhejiang University, Hangzhou, China; Health Canada

**Keywords:** *Salmonella*, whole-genome sequencing, antimicrobial resistance, poultry, serovar Enteritidis, salmonellosis

## Abstract

Paratyphoid avian salmonellosis is considered one of the leading causes of poultry death, resulting in significant economic losses to poultry industries worldwide. In China, especially in Shandong province, the leading producer of poultry products, several recurrent outbreaks of avian salmonellosis have been reported during the last decade where the precise causal agent remains unknown. Moreover, the establishment of earlier and more accurate recognition of pathogens is a key factor to prevent the further dissemination of resistant and/or hypervirulent clones. Here, we aim to use whole-genome sequencing combined with *in silico* toolkits to provide the genomic features of the antimicrobial resistance and virulence potential of 105 regionally representative non-Pullorum/Gallinarum Salmonella isolates recovered from dead poultry between 2008 and 2019 in Shandong, China. Additionally, phenotypic susceptibility to a panel of 15 antibiotics representing 11 classes was assessed by the broth microdilution method. In this study, we identified eight serovars and nine multilocus sequence typing (MLST) types, with Salmonella enterica serovar Enteritidis sequence type 11 (ST11) being the most prevalent (84/105; 80%). Based on their phenotypic antimicrobial resistance, 77.14% of the isolates were defined as multidrug resistant (≥3 antimicrobial classes), with the detection of one S. Enteritidis isolate that was resistant to the 11 classes. The highest rates of resistance were observed against nalidixic acid (97.14%) and ciprofloxacin (91.43%), followed by ampicillin (71.43%), streptomycin (64.77%), and tetracycline (60%). Genomic characterization revealed the presence of 41 resistance genes, with an alarmingly high prevalence of *bla*_TEM-1B_ (60%), in addition to genomic mutations affecting the DNA gyrase (*gyrA*) and DNA topoisomerase IV (*parC*) genes, conferring resistance to quinolones. The prediction of plasmid replicons detected 14 types, with a dominance of IncFIB(S)_1 and IncFII(S)_1 (87.62% for both), while the IncX1 plasmid type was considered the key carrier of antimicrobial resistance determinants. Moreover, we report the detection of critical virulence genes, including *cdtB*, *rck*, *sodCI*, *pef*, and *spv*, in addition to the typical determinants for Salmonella pathogenicity island 1 (SPI-1) and SPI-2. Furthermore, phylogenomic analysis revealed the detection of three intra-farm and five inter-farm transmission events. Overall, the detection of Salmonella isolates presenting high antimicrobial resistance and harboring different critical virulence genes is of major concern, which requires the urgent implementation of effective strategies to mitigate non-Pullorum/Gallinarum avian salmonellosis.

**IMPORTANCE** Avian salmonellosis is one of the leading global causes of poultry death, resulting in substantial economic losses in China (constituting 9% of overall financial losses). In Shandong province, a top poultry producer (30% of the overall production in China, with 15% being exported to the world), extensive outbreaks of avian salmonellosis have been reported in the past decade where the causal agents or exact types remain rarely addressed. From approximately 2008 to 2019, over 2,000 Salmonella strains were isolated and identified from dead poultry during routine surveillance of 95 poultry farms covering all 17 cities in Shandong. Approximately 1,500 isolates were confirmed to be of non-Pullorum/Gallinarum Salmonella serovars. There is an urgent need to understand the mechanisms behind the implication of zoonotic Salmonella serovars in systemic infections of poultry. Here, we analyzed populations of clinically relevant isolates of non-Pullorum/Gallinarum Salmonella causing chicken death in China by a whole-genome sequencing approach and determined that antimicrobial-resistant Salmonella Enteritidis remained the major cause in the past decades.

## INTRODUCTION

Poultry is an essential source for covering human nutritional needs and supplying required animal proteins to a vast population across religions. In recent decades, the continually growing population has increased the demand for meat products, especially poultry products, which has led to the development and intensification of poultry farming practices. In fact, advances in selective breeding and husbandry have significantly increased productivity in the last 50 years to meet the growing demand for poultry products (1). However, the transition to modern intensive farming practices has been challenging for farmers and accompanied by several issues related to animal health and welfare and sustainable food production, in addition to the concerns regarding emerging zoonotic pathogens (2).

Avian salmonellosis is an acute systemic infection caused by bacteria of the genus Salmonella. It is considered a major cause of avian morbidity and mortality worldwide, leading to the losses of millions of dollars to poultry industries (3). Salmonella enterica is a Gram-negative, non-spore-forming, rod-shaped bacterium belonging to the *Enterobacteriaceae* family and consists of over 2,600 serovars (4). Regarding the etiology, different types of avian salmonellosis have been reported, including pulorosis caused by S. enterica serovar Pullorum, typhus caused by *S.* Gallinarum, and paratyphoid caused by numerous other non-host-adapted serovars (5). Although avian salmonellosis is often linked to *S.* Pullorum and *S.* Gallinarum, which have been well documented in a few countries, including China, there are increasing cases of Salmonella strains other than *S*. Pullorum/Gallinarum involved in multiple cases of chicken death (6–8). This indicates that more attention should be paid to the recognition of avian salmonellosis with strong zoonotic potential.

In addition to the implementation of successful control programs and biosecurity measures as well as the development of numerous vaccines, antimicrobials have been used as first-line tools to prevent and/or mitigate avian diseases, including salmonellosis (9, 10). However, the long period of use of antimicrobials in poultry farms for several benefits, including treating infections (therapeutic), preventing infections (prophylaxis), and promoting growth (growth promoters) (11, 12), has led to the development of resistance to the critical antimicrobials used in both veterinary and human medicine, which threatens global health care systems (13). Recently, many studies have reported the emergence of resistance to multiple antimicrobials, including those belonging to the (fluoro)quinolone, 3rd- and 4th-generation cephalosporin, carbapenem, and polymyxin classes, in many pathogenic bacteria (14–18). Therefore, infections with these antimicrobial-resistant bacteria would likely lead to an increase in serious therapeutic failures, which is alarming regarding the lack of efficacy of the currently available treatment options.

Hence, the accurate and comprehensive detection of antimicrobial resistance (AMR) in poultry farms and monitoring of the spread and dissemination of these concerning clones provide crucial data, which can form the basis for the targeted treatment of diseased birds and the implementation of preventative measures. In fact, conventional detection based on phenotypic assessment provides only one facet of AMR features, while the genomic background encoding different AMR determinants with their major mechanisms remains undetermined. Today, the development of advanced sequencing technologies has encouraged the use of whole-genome sequencing (WGS) as a cost-effective method to provide the complete genetic determinants responsible for major mechanisms of antimicrobial resistance and virulence. More importantly, WGS supports the sharing of this information under the same standards within and between countries for further comparative purposes, improving control measures in a global sense (19–22).

In China, the poultry sector has experienced vigorous growth in the last 2 decades, in terms of both poultry numbers and the levels of output per bird. Eastern China, especially Shandong province, is considered the leading producer of poultry and eggs with the establishment of many giant poultry farms, from which a major part of poultry production is supplied to other neighboring provinces and East Asian countries, i.e., South Korea and Japan. In the last decades, several cases of recurrent poultry disease outbreaks have been recorded in Shandong province, in which ~2,000 Salmonella strains were isolated from dead poultry, and most of them belonged to non-Pullorum and non-Gallinarum Salmonella serovars. Hence, there is an urgent need to understand the mechanisms by which Salmonella, especially non-Pullorum/Gallinarum strains, cause diseases in poultry and resist different antimicrobial agents. In this regard, we conducted this study to provide the genomic features of the antimicrobial resistance and virulence of non-Pullorum/Gallinarum Salmonella isolates recovered from dead poultry to update the knowledge and, importantly, offer evidence-based practice for adjusting control policies accordingly.

## RESULTS

### Characterization of the examined Salmonella isolates.

**FIG 1:**
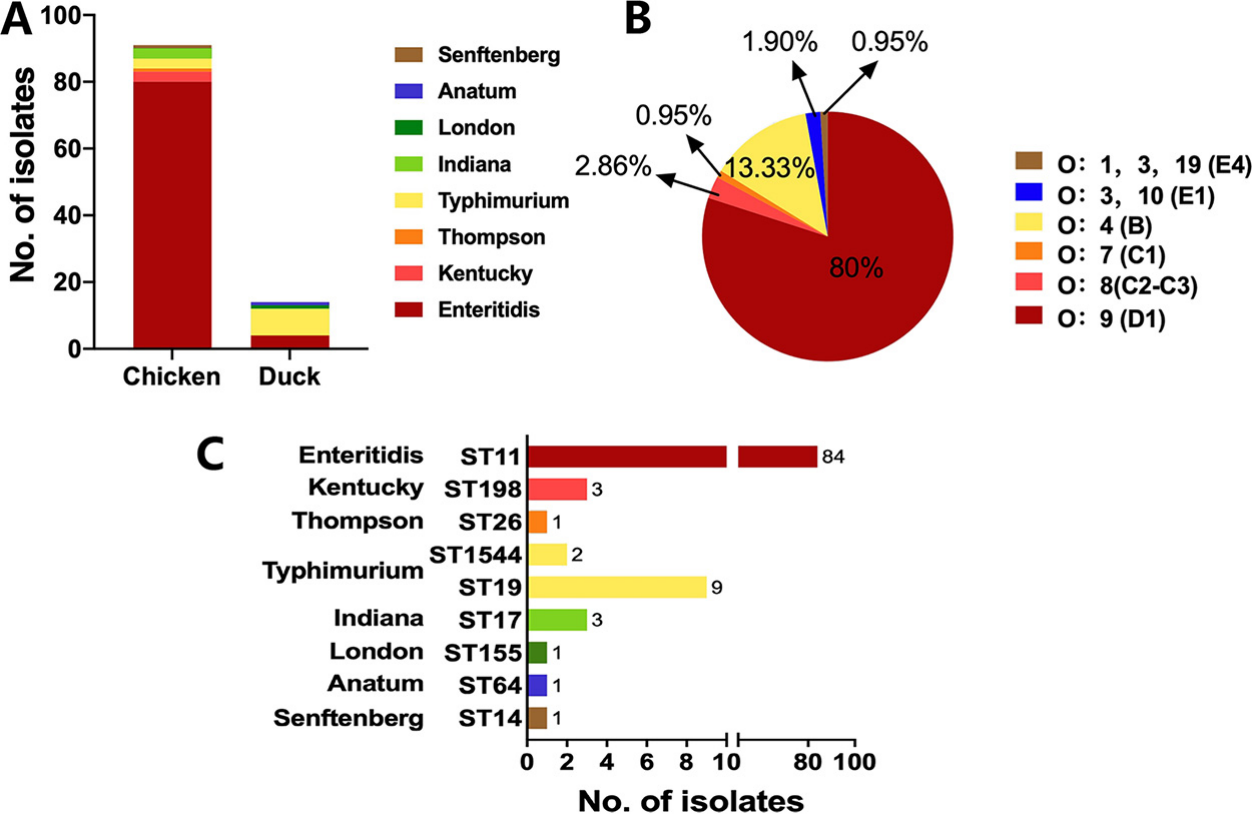
Distribution of serovars, serogroups, and MLST types among the studied Salmonella isolates. (A) The dominant serovar was S. Enteritidis. (B) The dominant serogroup was O:9 (D1). (C) The dominant MLST type was ST11.

Our results showed that the 105 Salmonella isolates were differentiated into eight different serovars (see Table S1 in the supplemental material), including Salmonella serovars Enteritidis (80%; 84/105), Typhimurium (10.48%; 11/105), Kentucky (2.86%; 3/105), Indiana (2.86%; 3/105), Thompson (0.9%; 1/105), London (0.9%; 1/105), Anatum (0.9%; 1/105), and Senftenberg (0.9%; 1/105) (Fig. 1A and Table 1), which were grouped into six different serogroups, including O:9 (D1) (80%; 84/105), O:4 (B) (13.33%; 14/105), O:8 (C2-C3) (2.86%; 3/105), O:3,10 (E1) (1.90%; 2/105), O:1,3,19 (E4) (0.95%; 1/105), and O:7 (C1) (0.95%; 1/105) (Fig. 1B and Table 2), while multilocus sequencing typing (MLST) analysis differentiated nine different types, including sequence type 11 (ST11) (80%; 84/105), ST19 (8.57%; 9/105), ST198 (2.86%; 3/105), ST17 (2.86%; 3/105), ST1544 (1.90%; 2/105), ST26 (0.9%; 1/105), ST155 (0.9%; 1/105), ST64 (0.9%; 1/105), and ST14 (0.9%; 1/105) (Fig. 1C and Table 2).

**TABLE 1:** Distribution of the studied Salmonella isolates

Salmonella serovar	No. of isolates from animal		Total no. of isolates
	Chicken	Duck	
Senftenberg	1	0	1
Anatum	0	1	1
London	0	1	1
Indiana	3	0	3
Typhimurium	3	8	11
Thompson	1	0	1
Kentucky	3	0	3
Enteritidis	80	4	80

Total	91	14	105

**TABLE 2:** Allelic profiles and MLST types of the studied Salmonella isolates

Serogroup	Salmonella serovar	MLST pattern (no. of isolates)	Allelic type						
			*aroC*	*dnaN*	*hemD*	*hisD*	*purE*	*sucA*	*thrA*
O:1,3,19 (E4) (*n* = 1)	Senftenberg	ST14 (1)	7	6	8	8	7	8	13

O:3,10 (E1) (*n* = 2)	Anatum	ST64 (1)	10	14	15	31	25	20	33
	London	ST155 (1)	10	60	58	66	6	65	16

O:4 (B) (*n* = 14)	Indiana	ST17 (3)	8	8	11	11	5	11	15
	Typhimurium	ST19 (9)	10	7	12	9	5	9	2
		ST1544 (2)	10	7	12	230	5	9	2

O:7 (C1) (*n* = 1)	Thompson	ST26 (1)	14	13	18	12	14	18	1

O:8 (C2-C3) (*n* = 3)	Kentucky	ST198 (3)	76	14	3	77	64	64	67

O:9 (D1) (*n* = 84)	Enteritidis	ST11 (84)	5	2	3	7	6	6	11

### Antimicrobial resistance phenotype and related genetic determinants.

**FIG 2:**
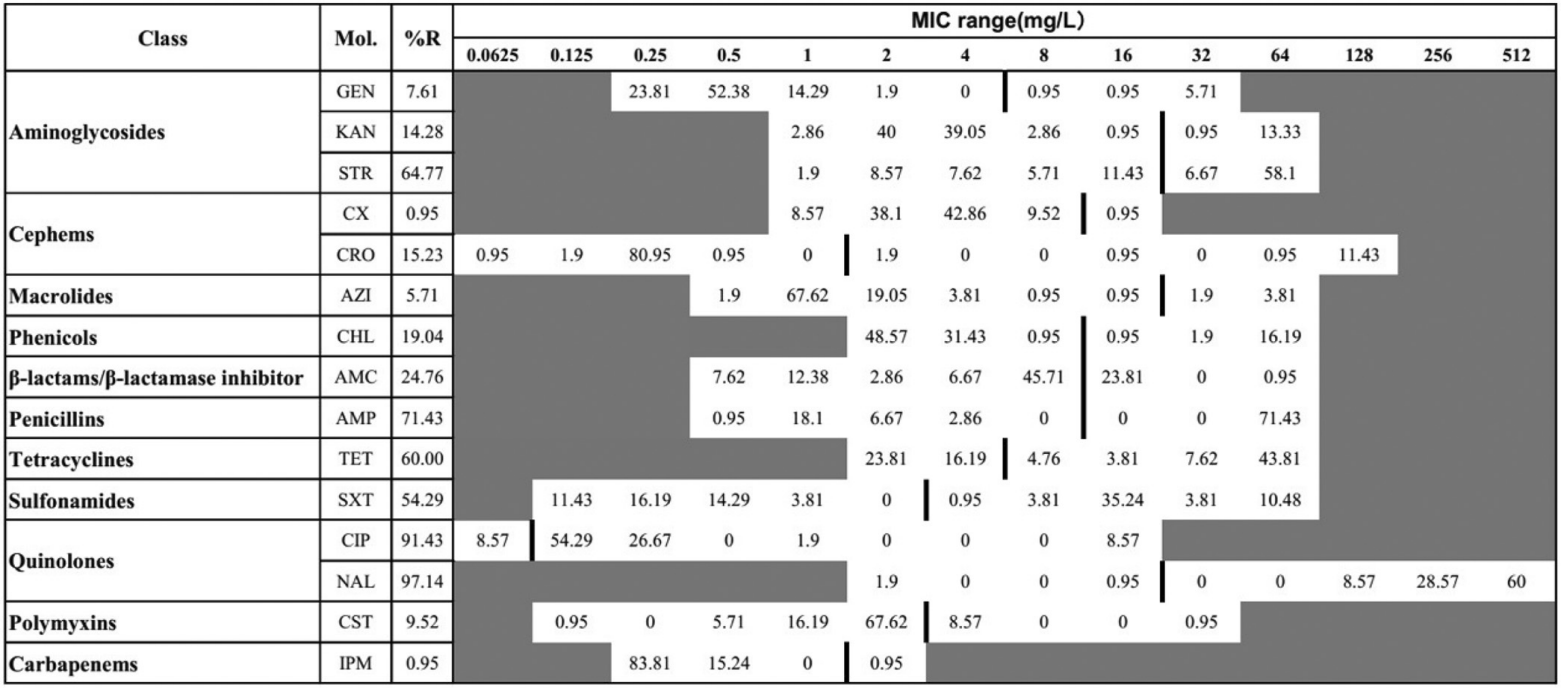
Distribution of MIC values among the examined Salmonella isolates (*n* = 105) against the tested antimicrobial agents. The tested antimicrobial agents in the second column are as follows: gentamicin (GEN), kanamycin (KAN), streptomycin (STR), cefoxitin (CX), ceftriaxone (CRO), azithromycin (AZI), chloramphenicol (CHL), amoxicillin-clavulanic acid (AMC), ampicillin (AMP), tetracycline (TET), trimethoprim-sulfamethoxazole (SXT), ciprofloxacin (CIP), nalidixic acid (NAL), colistin (CST), and imipenem (IPM). The values in each cell show the percentage of isolates corresponding to each MIC dilution level. The vertical bar indicates the cutoff level of the MIC for each antimicrobial agent at the highest value of that particular cell’s dilution for susceptibility (equal to or less than) and resistance (greater than). For better clarification and ease of analysis, the results of intermediate values were combined with those of resistance. %R, percent resistant.

**FIG 3:**
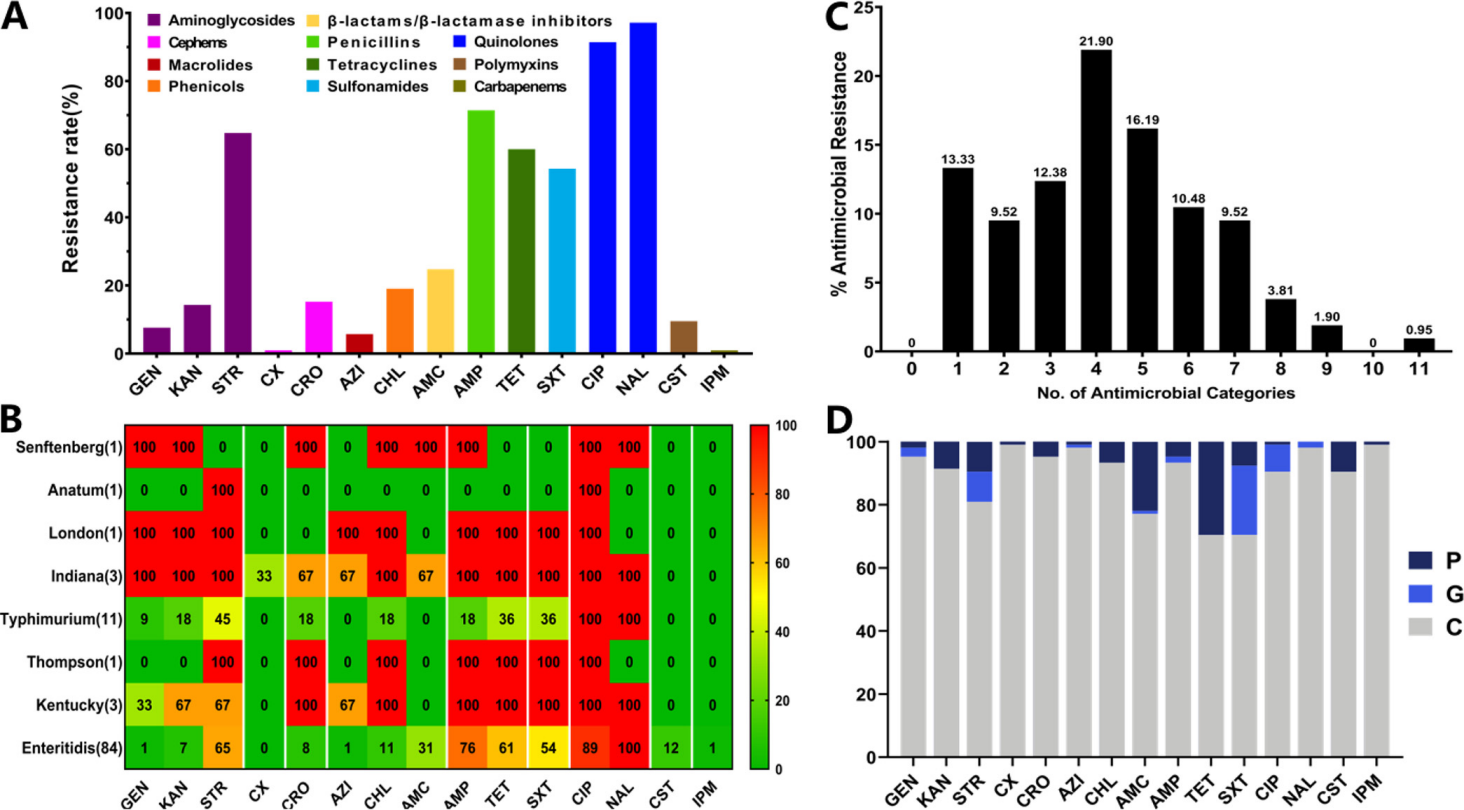
Antimicrobial resistance of the studied Salmonella isolates. (A) Prevalence of antimicrobial resistance against 15 antibiotics belonging to 11 classes. The same category of antibiotics is represented by the same color. (B) Distribution of antimicrobial resistance (percentages) among different serovars. (C) Distribution of multidrug resistance patterns among the studied isolates. (D) Concordance (percentages) between phenotypic and genotypic antimicrobial resistance. C, concordance (genotype positive/phenotype positive or genotype negative/phenotype negative); G, genotype positive/phenotype negative; P, phenotype positive/genotype negative.

Our findings registered especially frequent resistance to nalidixic acid (NAL) (97.14%) and ciprofloxacin (CIP) (91.43%), followed by ampicillin (AMP) (71.43%), streptomycin (STR) (64.77%), and tetracycline (TET) (60%), while the lowest levels of resistance were observed against cefoxitin (CX) (0.95%) and imipenem (IPM) (0.95%) (Figs. 2 and 3A). Additionally, despite the low numbers of isolates of each serovar detected (except S. Enteritidis and *S.* Typhimurium), which influence comparability between serovars, it appears that *S*. Indiana possessed the highest levels of resistance to different antimicrobial agents, except colistin (CST) and imipenem (Fig. 3B). Moreover, we reported 43 different AMR patterns where resistance to three or more antimicrobial classes (multiple-drug resistance [MDR]) was detected in 77.14% (81/105) of the studied isolates (Fig. 3C), in which the predominant MDR phenotypic patterns were “aminoglycosides plus penicillins plus quinolones” (58.09%; 61/105), followed by “penicillins plus quinolones plus tetracyclines” (51.43%; 54/105) and “aminoglycosides plus quinolones plus tetracyclines.” Importantly, we noted the detection of one S. Enteritidis isolate resistant to all 11 antimicrobial classes (Table S2).

**FIG 4:**
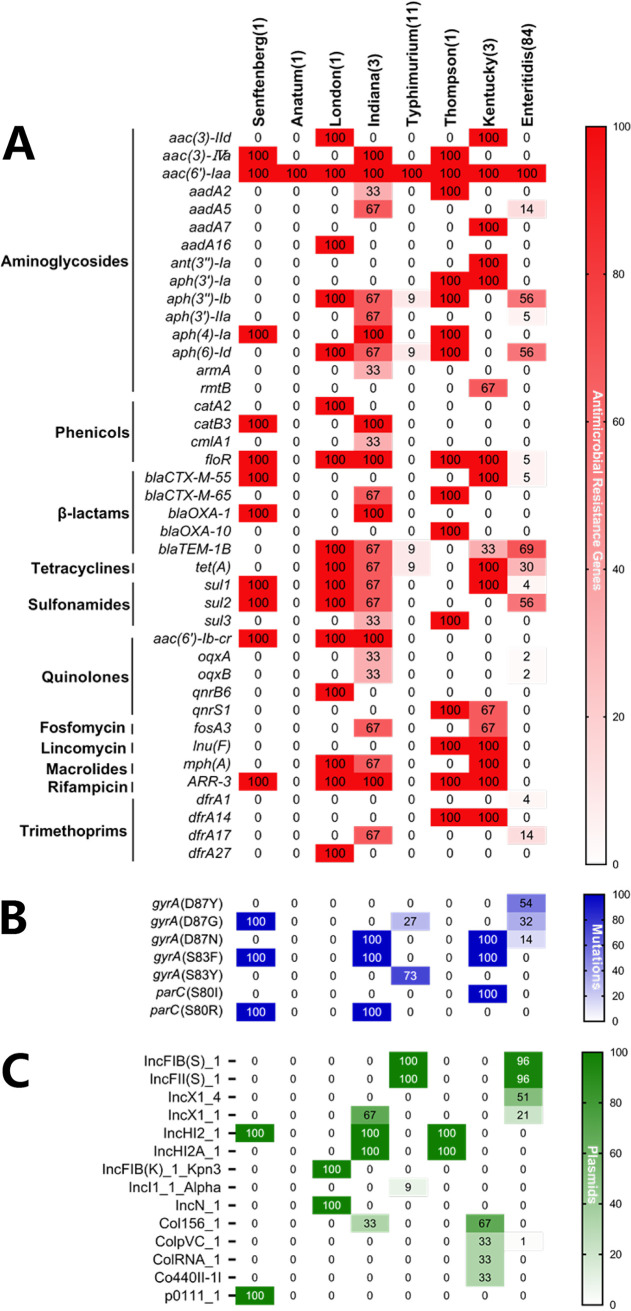
Prevalences of antimicrobial resistance determinants (A), genomic mutations (B), and plasmid replicons (C) among serovars. The number in each cell corresponds to the percent prevalence of each element.

Our results showed the detection of 41 different genes encoding resistance to 11 antimicrobial classes (Table S1); among them, the gene *aac(6*′*)-Iaa*, encoding resistance to aminoglycosides, was detected in all isolates (100%) (Fig. 4A). Regarding the distribution of serovars, our results showed that *S*. Indiana possesses a higher number of antimicrobial resistance genes than the other serovars. Additionally, screening of genomic mutations showed the detection of double mutations in the *gyrA* gene and single mutations in the *parC* gene, which are responsible for the high rates of resistance to quinolones, in isolates belonging to Salmonella serovars Kentucky, Indiana, and Senftenberg. In contrast, single and double mutations that are responsible for moderate resistance to quinolones have been detected in the *gyrA* gene in isolates of Salmonella serovars Typhimurium and Enteritidis (Fig. 4B and Table S1).

The concordance between phenotypic AMR and the presence of the corresponding genetic determinants, including chromosomal mutations, has been evaluated, and the obtained results are summarized in Fig. 3D. Our findings showed that the highest concordance was observed for cefoxitin (99.5%) and imipenem (99.5%), while the lowest concordance was observed for tetracycline (70.48%) and trimethoprim-sulfamethoxazole (SXT) (70.48%), of which 29.52% of isolates showed phenotypic resistance to tetracycline but without carrying the corresponding genetic determinants. However, 21.9% of isolates carried genetic determinants encoding resistance to folate pathway antagonists but without exhibiting phenotypic resistance to the combination of trimethoprim and sulfamethoxazole.

### Prediction of virulence genes.

**FIG 5:**
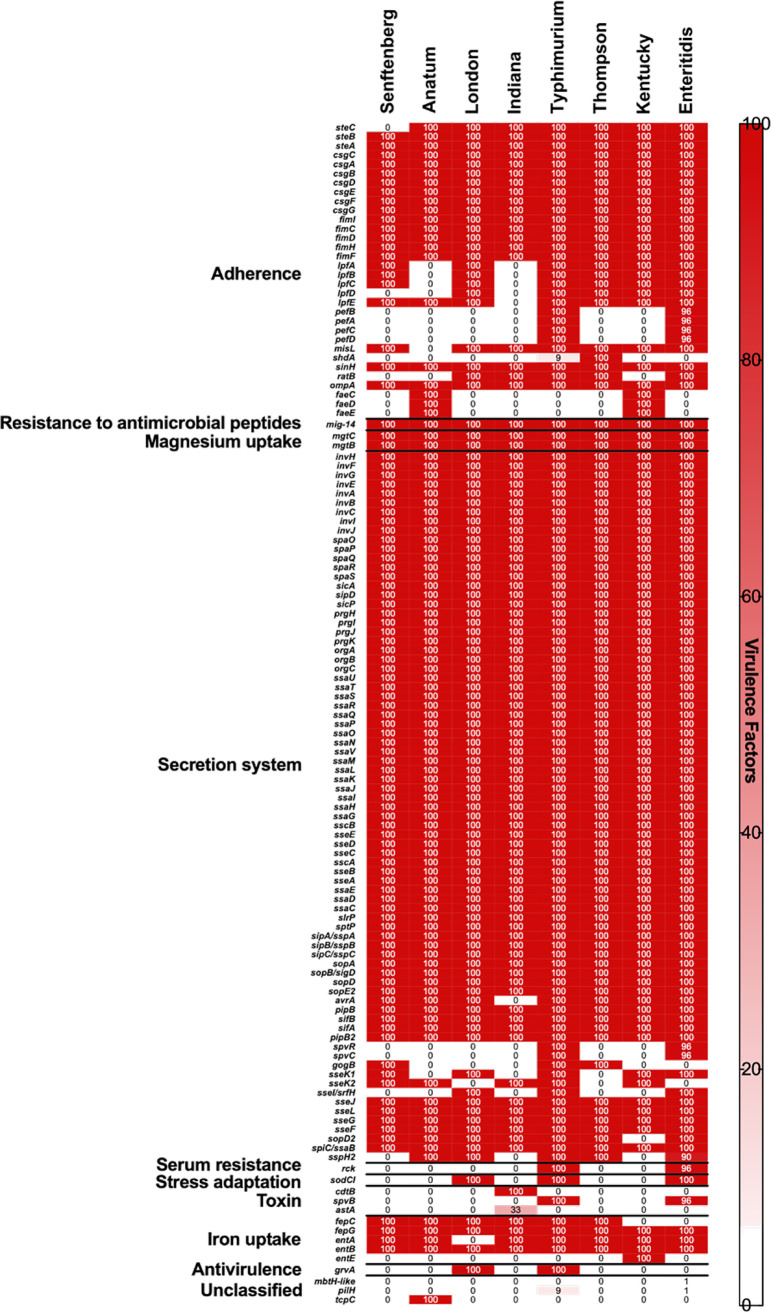
Distribution of virulence genes among the studied isolates. The color of the individual cells varies with the percent prevalence of each virulence gene.

Our findings showed the detection of 126 different virulence genes representing different virulence and pathogenicity mechanisms of Salmonella (Fig. 5). Additionally, our results demonstrate the presence of the *cdtB* gene encoding typhoid toxin production and the *astA* gene (EAST1 toxin) in isolates belonging to *S*. Indiana with prevalences of 100% (3/3) and 33% (1/3), respectively. Moreover, the *rck* gene, encoding a serum resistance protein, was detected in *S*. Typhimurium (100%) and S. Enteritidis (96%), and the *sodCI* gene, encoding an enzyme that protects Salmonella from the oxidative burst in the macrophage environment, was detected in all isolates of Salmonella serovars London, Typhimurium, and Enteritidis. However, the *spv* locus that gene cluster (*spvR*, *spvC*, and *spvB*), implicated in the virulence system of nontyphoid Salmonella, and the *pef* genes (*pefA*, *pefB*, *pefC*, and *pefD*), encoding fimbriae, were detected in all isolates of *S.* Typhimurium (100%) and the majority of S. Enteritidis isolates (96%). On the other hand, all of the studied Salmonella isolates carried the typical virulence genes of Salmonella pathogenicity island 1 (SPI-1) and SPI-2 (Table S1).

### Plasmid replicons.

The prediction of plasmid replicons in the studied Salmonella isolates reveals the detection of 14 different plasmids (Fig. 4C), of which the plasmid types IncFIB(S)_1 and IncFII(S)_1 were the most prevalent among the studied isolates. When comparing the distributions between serovars, our results showed that S. Enteritidis harbors the most diversified plasmids (five different plasmid types), followed by *S*. Kentucky and *S*. Indiana (four different plasmid types each), while no plasmid replicon was detected in *S*. Anatum. Additionally, the plasmid type IncHI2_1 was detected in three different serovars, including Indiana, Thompson, and Senftenberg (Table S1).

### Cooccurrence of plasmid contigs and antimicrobial resistance determinants.

The cooccurrence of plasmid contigs and antimicrobial resistance determinants was evaluated, and the results are presented in Table S3. Our results showed that the IncX1 plasmid type is likely the key carrier of antimicrobial resistance determinants, in addition to the IncFIB(S) and IncFII(S) plasmids. The AMR determinants most associated with IncX1 are *bla*_TEM-1B_, *sul1*, *sul2*, *aph(3*″*)-Ib*, *aph(6)-Id*, *aph(3*′*)-IIa*, *ant(3*″*)-Ia*, *aadA5*, *dfrA17*, *dfrA1*, and *tet*(*A*), encoding resistance to the β-lactam, sulfonamide, aminoglycoside, trimethoprim, and tetracycline classes. Notably, the observed plasmid and antimicrobial resistance gene (ARG) cooccurrences were detected mainly in S. Enteritidis (70.23%; 59/84), and cooccurrence between IncX1 and *aph(3*′*)-IIa_2* was detected in 2 out of 3 *S*. Indiana isolates. Nevertheless, several contigs in S. Enteritidis isolates were suggested to contain multiple plasmid replicons [IncFIB(S)_1, IncX1_4, and IncFII(S)_1].

### Phylogenetic analysis and transmission events.

**FIG 6:**
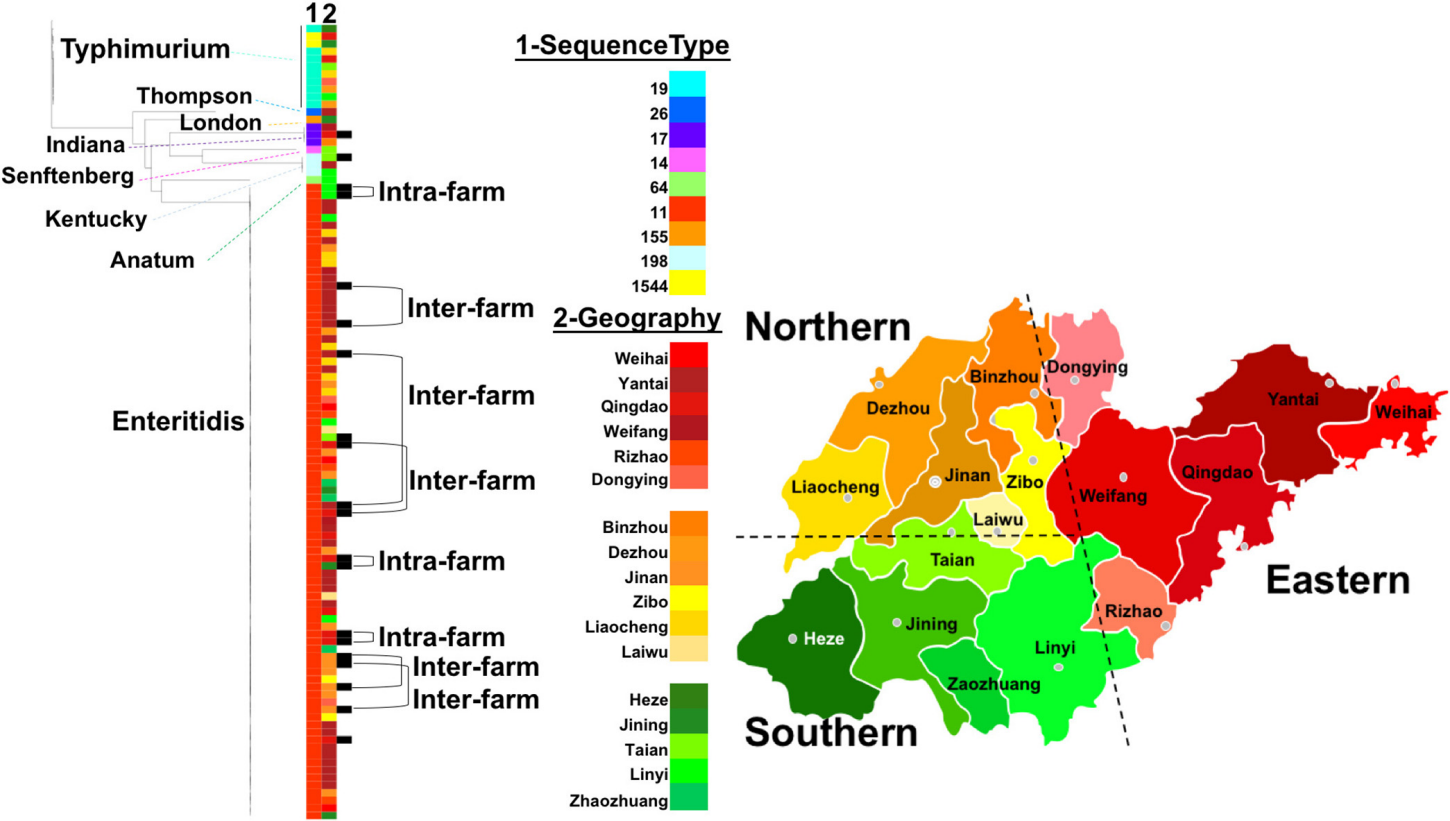
Phylogenomic tree of the examined Salmonella isolates based on core single-nucleotide polymorphisms (SNPs). The phylogenetic relationship was built based on ~5,000 core SNPs, and the heatmap is colored with individual isolates by sequence type and place of sampling. For ease of analysis, we divide the Shandong province into three regions, Eastern (Weihai, Yantai, Qingdao, Weifang, Rizhao, and Dongying), Northern (Binzhou, Dezhou, Jinan, Zibo, Liaocheng, and Laiwu), and Southern (Heze, Jinning, Taian, Linyi, and Zhaozhuang). Intrafarm association was defined as isolates with a relatively close genetic relationship and isolates from the same farm. Interfarm association was defined as isolates from different farms but within the same geographic regions, the suggested Eastern, Northern, or Southern part.

To investigate the genomic relationships among the studied isolates and the sampling farms, we elaborated a phylogenomic tree based on core single-nucleotide polymorphism (SNP) analysis (Fig. 6). Our results showed that the isolates belonging to the same serovars and with similar sequence types are grouped close to each other, and all Salmonella Enteritidis isolates belong exclusively to ST11. The remaining inter-farm and intra-farm transmissions were examined only among isolates of S. Enteritidis.

To further evaluate possible transmission events, we coded the farms in the same city with one color (defined as intra-farm association events) and grouped geographically related farms into three major parts (Northern, Eastern, and Southern), defining them as inter-farm association events. Based on phylogenomic relationships, we found three pairs of isolates with intra-farm associations and five pairs of isolates with interfarm associations (Fig. 6).

## DISCUSSION

Poultry farming is the leading supplier of animal proteins to a wide population, including China. According to newly available data, more than 60 billion chickens are consumed annually (23), highlighting the importance of chicken consumption in the diet of populations across the world. At the same time, poultry farms are considered a favorable biotope for the development of many pathogens like Salmonella, causing huge economic losses for producers (6, 8, 18, 24). Hence, to provide in-depth data about the AMR and virulence of Salmonella isolates causing poultry death, we used WGS combined with accurate phenotypic assays to analyze a collection of 105 Salmonella isolates recovered from dead poultry between 2008 and 2019, in 95 farms representing all 17 cities of Shandong province.

The determination of serovars and MLST patterns based on WGS showed the dominance of S. Enteritidis ST11 among the identified serovars. Similarly, Elbediwi et al. reported that S. Enteritidis is the most frequent serovar in dead chick embryos in Henan province in Central China (6). Additionally, Wang et al. reported the dominance of S. Enteritidis in isolates recovered from dead-in-shell chicken embryos in Northern China (25). Furthermore, a study performed by the Walker group demonstrated the implication of S. Enteritidis in persistent infections in broiler chickens in North Carolina (24). Generally, avian salmonellosis has been linked for decades to isolates of *S*. Gallinarum (biovars Gallinarum and Pullorum), which have been eradicated in many developed countries after the establishment of different mitigation strategies (26). Thus, paratyphoid diseases caused by zoonotic Salmonella serovars, especially S. Enteritidis, have taken place among a major form of salmonellosis in poultry (24). The traditional *S*. Pullorum dominance phenomena could start to change in certain regions of China (18) considering that paratyphoid Salmonella rarely causes acute clinical disease except in very young birds exposed to stressful conditions. The high death rate of paratyphoid Salmonella is likely due to a cooccurrence of numerous immunosuppressed pathogens, i.e., infectious bursal disease virus circulation in China, together with the transition of farming practices to high stocking densities in giant poultry farms (27), which is worthy of further investigations.

In this study, the susceptibilities of the examined Salmonella isolates to 15 antibiotics belonging to 11 different classes were evaluated, and the results showed that 77.14% of the isolates were resistant to at least three different classes of antimicrobials (considered MDR). This was a higher percentage than those reported for isolates recovered from diseased birds in Northern China (25); dead chick embryos in Henan province, China (6); and dead-in-shell chicken embryos in Shandong province, China (8). Additionally, we reported high rates of resistance to nalidixic acid (97.14%) and ciprofloxacin (91.43%). In fact, quinolone resistance, especially to the first generation (nalidixic acid), has become a common issue in China and elsewhere (24, 25, 28). It has been noted that feed supplemented with quinolones has been widely used in poultry farms in China (29), which might exert selective pressure on pathogens, allowing the development of quinolone resistance in many Salmonella serovars. In fact, our findings are alarming to public health care systems since antimicrobials belonging to the quinolone category are used as the front-line choice to treat clinical cases of human salmonellosis. Hence, there is a need to establish strict policies in order to ban the use of critically important antibiotics in farm animals.

To provide in-depth data about the mechanisms behind the phenotypic resistance of the studied isolates, we assessed the presence of different ARGs based on WGS analysis, and the results showed the detection of 41 different genes encoding resistance to 11 antimicrobial classes. The *aac(6′)-Iaa* gene, encoding resistance to aminoglycosides, was detected in all of the studied isolates (100%), which was in agreement with previous Chinese and South Korean studies (6, 30, 31). However, another Chinese study reported the dominance of *bla*_TEM_ in Salmonella isolates recovered from dead-in-shell chicken embryos in Shandong province (8). The high level of resistance to quinolones detected in this study may be explained by the acquisition of plasmid-mediated quinolone resistance (PMQR) genes as horizontally transferable elements as well as point mutations in the quinolone resistance-determining region (QRDR) affecting the DNA gyrase and DNA topoisomerase IV genes (32–34). In this regard, we report the detection of the *aac(6′)-Ib-cr*, *oqxA*, *oqxB*, *qnrB6*, and *qnrS1* genes encoding decreased susceptibility to quinolones in isolates of *S*. Senftenberg, *S*. London, *S*. Indiana, *S*. Thompson, *S*. Kentucky, and S. Enteritidis. These genes are carried on mobile elements like plasmids, which facilitate the horizontal dissemination of quinolone determinants and resistance between bacterial species. In addition, the assessment of genomic mutations in the QRDRs revealed different punctual mutations in the *gyrA* and *parC* genes; we found that all isolates of *S*. Senftenberg, *S*. Indiana, and *S*. Kentucky had double mutations in the *gyrA* gene and single mutations in the *parC* gene. This might explain the high levels of resistance of these isolates to quinolones, while most S. Enteritidis and *S.* Typhimurium isolates presented mutations only in the *gyrA* gene. Note that *S*. Anatum, *S*. London, and *S*. Thompson did not harbor any mutation in the QRDRs. Indeed, it has been demonstrated that isolates with double mutations in the *gyrA* gene and a single mutation in the *parC* or *parE* gene were highly resistant to quinolones, while isolates that presented a single mutation in the *gyrA* or *parC* gene showed only reduced susceptibility (intermediate), especially to ciprofloxacin (35).

The concordance between phenotypic and genotypic resistance showed high coherence, especially for cefoxitin and imipenem, while moderate coherence was found for tetracycline and trimethoprim-sulfamethoxazole. Our findings are in accordance with those reported previously for Salmonella isolates from a pig slaughterhouse in Hangzhou, China (36). Similarly, a large-scale study reported high coherence between phenotypic and genotypic resistance for all of the tested antibiotics (37). It has been noted that resistance to quinolones is not limited to the acquisition of resistance genes carried on mobile genetic elements, but clinical quinolone resistance is associated with stable genomic mutations affecting the DNA gyrase and DNA topoisomerase IV genes (35). Moreover, some mechanisms like coresistance and cross-resistance may confer phenotypic resistance to an antibiotic without the detection of the corresponding genetic elements; on the other hand, not all detected genes may induce phenotypic resistance (19, 37–39). Regarding the combination of trimethoprim and sulfamethoxazole in particular, it should be noted that the presence of a single gene mediating resistance to either substance might not render an isolate resistant to the combination of both substances, which may influence the calculated concordance between phenotype and genotype.

The prediction of virulence genes revealed the detection of different genes implicated in the pathogenicity mechanisms of Salmonella spp. in addition to the typical genes of SPI-1 and SPI-2. The *cdtB* gene, encoding typhoid toxin production, and the *astA* gene (EAST1 toxin) were identified only in *S*. Indiana, which agrees with previous studies reporting the association of the *cdtB* gene with *S*. Indiana isolates circulating in China (6, 30). Importantly, the *cdtB* gene was identified in nontyphoidal Salmonella isolates causing invasive infections in humans (40, 41). In contrast, EAST1 toxin is associated with diarrheal disease in humans and various animal species, including cattle, swine, and poultry (42). The serum resistance gene *rck* that encodes an outer membrane protein that enhances bacterial adhesion and invasion and also confers high-level resistance to antibacterial agents, including antibiotics and biocides (43), was detected in *S*. Typhimurium and S. Enteritidis. The *sodCI* gene, involved in the protection of Salmonella from the oxidative burst inside macrophages (44, 45), was detected in Salmonella serovars London, Typhimurium, and Enteritidis, whereas the *spv* locus that clustered the *spvR*, *spvC*, and *spvB* genes was detected in *S*. Typhimurium and S. Enteritidis. This locus plays an essential role in the virulence system of nontyphoid Salmonella, and its abilities to deteriorate innate immune function, inhibit the responses of neutrophils and macrophages during infection, as well as restrain the formation of autophagosomes induced by Salmonella have been demonstrated (46). Furthermore, *S*. Typhimurium and S. Enteritidis isolates carried *pef* genes (*pefA*, *pefB*, *pefC*, and *pefD*) encoding fimbriae. It has been reported that the *pef* fimbrial operon mediates adhesion to the murine small intestine (47) and has a role in cecal colonization by S. Enteritidis during infection of chickens (48).

The potential for the rapid dissemination of resistance genes and virulence factors between isolates is the leading obstacle in limiting the international spread of MDR pathogens. Plasmids are considered the main mobile elements responsible for the horizontal transfer of genetic determinants. In fact, the prediction of plasmid replicons showed the detection of 14 different plasmids, with a dominance of IncFIB(S)_1 and IncFII(S)_1, which were detected only in Salmonella serovars Typhimurium and Enteritidis. Interestingly, it has been reported that these plasmid types carry antimicrobial resistance genes, *pef* (plasmid encoding fimbriae) (49), and the *spv* virulence-associated region (50), and they confer hypervirulence and contribute to bacterial fitness (51). However, *S*. Indiana, which was considered the most resistant serovar in this study, carries plasmid types IncX1_1, IncHI2_1, IncHI2A_1, and Col156_1; these plasmids have been associated with the acquisition of resistance to different classes of antimicrobials, including aminoglycosides, sulfonamides, tetracyclines, quinolones, polymyxins, and β-lactams (52, 53). Furthermore, our findings demonstrated that the plasmid type IncX1, in addition to IncFII(S) and IncFIB(S), is the key carrier of antimicrobial resistance determinants in Salmonella, which is in agreement with previous findings from China and elsewhere (54–57). Hence, the presence of these plasmids in the studied isolates is of major concern and may mediate the horizontal transmission of antimicrobial resistance along the food chain.

The phylogenomic relationship analysis showed that isolates of the same serovar and with similar sequence types were grouped close to each other, and farm-to-farm analysis suggested the detection of three intrafarm and five interfarm transmission events, which may explain the persistence of infection in certain farms where recurrent cases have been recorded for a long time. The likely intrafarm transmission event has been clearly demonstrated by the closest genetic relationship and the isolates from the same farm but different death cases. However, the exact interfarm transmission event can only be suggested as the same S. Enteritidis and ST11 and small evolutionary clades. As the large-scale transgeographical livestock trade is limited in China, we consider that only small regional-level trade is possible; therefore, intrafarm transmission happens only locally. Nevertheless, this may be due to nonadherence to strict sanitizing measure application on poultry farms, which allows the persistence of pathogens in the farming environment or vertical transmission between poultry generations (58). Moreover, supplying chicks from the same hatcheries or between farms could be another factor in the interfarm transmission risk. Hence, it is necessary to enhance hygienic and sanitizing measures as well as detection capabilities for Salmonella carriers on farms. Additional recording of the traceability of inputs and outputs on each farm could help to limit the persistence and dissemination of Salmonella in and between the poultry farms.

### Conclusion.

In this study, we used whole-genome sequencing followed by bioinformatic analysis to provide accurate information about the antimicrobial resistance and virulence of non-Pullorum/Gallinarum Salmonella strains isolated from dead poultry in Shandong province, China. We found that S. Enteritidis is the leading driver of poultry death in this province, and liver and embryo samples dominated most of the cases. Importantly, we showed a high prevalence of MDR isolates with the detection of mobile genetic determinants encoding resistance to different antibiotics in addition to genomic mutations encoding resistance to quinolones. Moreover, we report the detection of critical virulence genes like *cdtB*, encoding typhoid toxin production; *rck*, encoding serum resistance; *sodCI*, involved in the protection of Salmonella from the oxidative burst inside macrophages; and the genes clustered in the *spv* locus and the *pef* operon, in addition to the typical genes of SPI-1 and SPI-2. Therefore, the detection of multidrug-resistant and hypervirulent Salmonella isolates is alarming for both animal and human health, which requires the urgent application of new policies to deal with non-Pullorum/Gallinarum Salmonella infections in poultry farms. In the end, we believe that the application of whole-genome sequencing for epidemiological analysis on animal farms, besides its application to clinical and zoonotic pathogens, with the possibility of sharing data, is an urgent requirement to deal with emerging MDR and hypervirulent pathogens.

## MATERIALS AND METHODS

### Sample collection.

**FIG 7:**
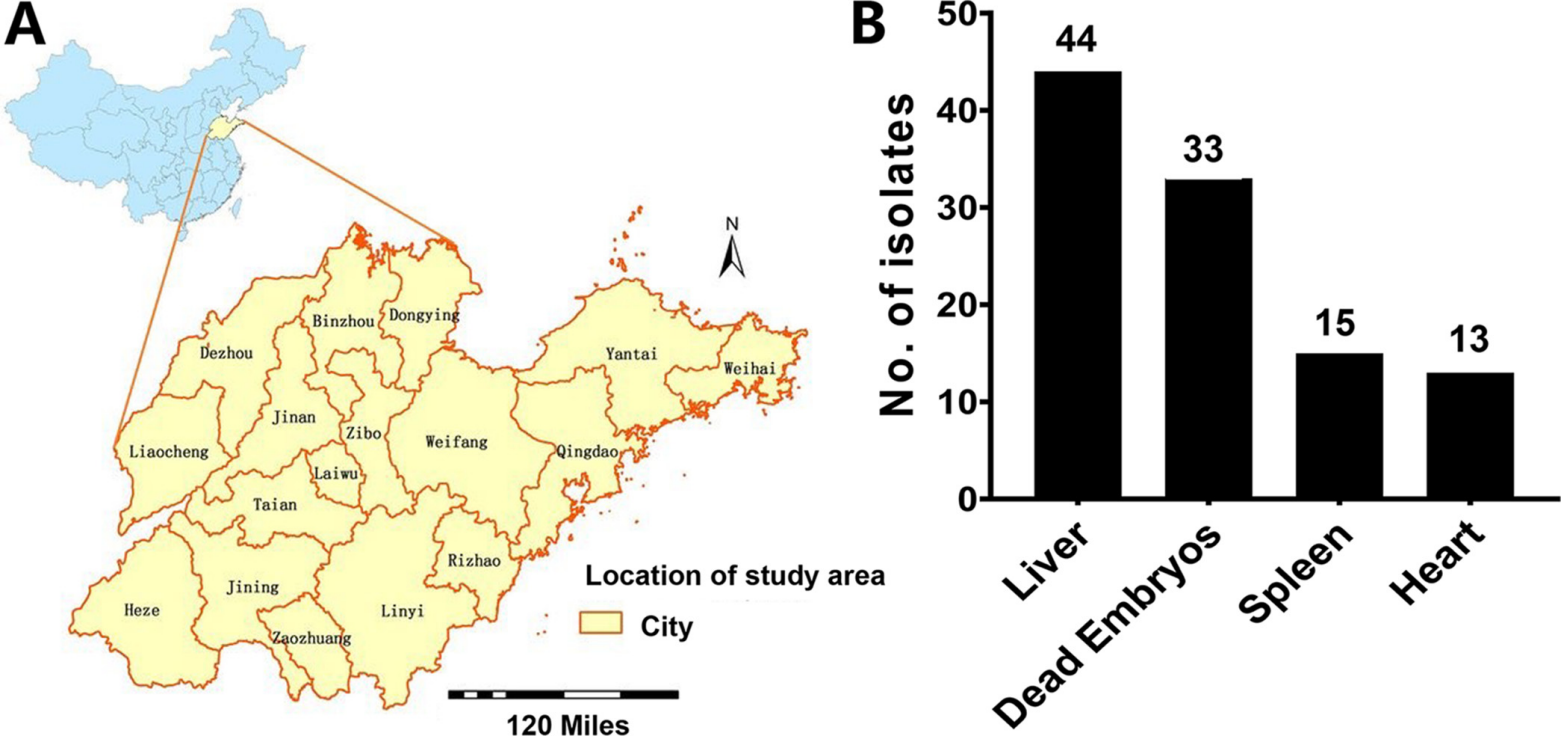
Distribution of sampling areas and sample origins. (A) The studied area, with the 17 cities of Shandong province represented. (B) Distribution of Salmonella isolates according to the origin/type of sample.

From 2008 to 2019, a total of ~2,000 Salmonella clinical isolates were collected from 95 poultry farms covering all 17 cities of Shandong province in China (Fig. 7A), in which ~1,500 isolates were identified as non-Pullorum/Gallinarum isolates. This is part of a new surveillance program directed by the Shandong Academy of Agricultural Sciences. In this study, we selected 105 of these Salmonella isolates (representing 1 to 2 isolates from each farm) originating from dead poultry (chicken, *n* = 91; duck, *n* = 14) according to the same sampling ratio of chickens to ducks as that in the original sample pool. These isolates were obtained from different types of samples, including dead embryos, liver, heart, and spleen (Fig. 7B). The studied isolates (*n* = 105) were selected based on different criteria, including (i) those that were clearly documented in death cases in either chick or embryo samples, (ii) those that could cover all regions or cities in Shandong province, and (iii) those that could recruit the largest number of farms that have documented avian salmonellosis. Sampling was conducted according to a method recommended by the World Organisation for Animal Health (OIE) terrestrial code (59). Importantly, note that these isolates are part of a new investigation in Shandong province in China and have not been documented previously.

### Isolation and characterization of Salmonella strains.

During this surveillance program, the isolation and identification of Salmonella were performed according to protocols described in previously published articles, with a few modifications (18, 30). Briefly, preenrichment of all samples was performed in buffered peptone water (BPW) at 37°C overnight, and selective enrichment was then performed on the preenriched cultures by inoculation on modified semisolid Rappaport-Vassiliadis (MSRV) agar at 41.5°C for 24 h. Finally, a loopful of material from MSRV agar was transferred to xylose-lysine-deoxycholate (XLD) agar and incubated at 37°C for 24 h. Red colonies with a black center (or, occasionally, translucent red in the case of H_2_S-negative strains) on XLD agar were presumed to be Salmonella. Afterward, the obtained presumed isolates were confirmed by the matrix-assisted laser desorption ionization–time of flight mass spectrometry (MALDI-TOF MS) technique (Bruker, USA) and the amplification of the *invA* gene by conventional PCR (30, 60). The confirmed isolates were serotyped by the slide agglutination method by using anti-O and anti-H sera (Bio-Rad Laboratories, Inc., USA). The serovar results were interpreted according to the Kauffmann-White-Le Minor scheme (61).

### Antimicrobial susceptibility test.

The antimicrobial susceptibilities of the 105 selected Salmonella isolates were evaluated by the determination of MIC values using the broth microdilution method. This assay was performed in 96-well plates, and after incubation overnight at 37°C, the growth of bacteria was determined by measuring the optical density at 600 nm with a microplate reader (Synergy H1; BioTek). Afterward, the obtained results were interpreted according to the criteria recommended by the Clinical and Laboratory Standards Institute (62), as described previously (63). Fifteen different antimicrobials belonging to 11 categories were used for this assay (Fig. 1): aminoglycosides (gentamicin [GEN], kanamycin [KAN], and streptomycin [STR]), cephems (cefoxitin [CX] and ceftriaxone [CRO]), macrolides (azithromycin [AZI]), phenicols (chloramphenicol [CHL]), β-lactam/β-lactamase inhibitor combinations (amoxicillin-clavulanic acid [AMC]), penicillins (ampicillin [AMP]), tetracyclines (tetracycline [TET]), sulfonamides (trimethoprim-sulfamethoxazole [SXT]), quinolones (ciprofloxacin [CIP] and nalidixic acid [NAL]), polymyxins (colistin [CST]), and carbapenems (imipenem [IPM]). Isolates with an MIC in the intermediate range for an antimicrobial agent were categorized as resistant for ease of analysis, and multiple-drug resistance (MDR) refers to antimicrobial resistance to at least three antimicrobial classes. Escherichia coli ATCC 25922 and Pseudomonas aeruginosa ATCC 27853 were used as quality control strains.

### DNA extraction and whole-genome sequencing.

Genomic DNA of all 105 selected Salmonella isolates was extracted from cultures incubated in Luria-Bertani (LB) broth overnight using a FastPure bacterial DNA isolation minikit (Vazyme Biotech Co., Ltd., Nanjing, China) according to the manufacturer’s instructions and quantified using a Nanodrop 1000 instrument (Thermo Fisher Scientific, USA). Genomic DNA of eligible quality was sequenced on the Illumina NovaSeq 6000 platform by Novogene (Beijing, China), as described previously (6).

### Bioinformatic analysis.

After the sequencing raw data (FASTQ file) were returned, all subsequent *in silico* analyses were performed on the in-house Galaxy platform as described previously (64). Briefly, the quality of sequencing and trimming was checked with the fastQC toolkit, while low-quality sequences and joint sequences were removed with Trimmomatic (65). The raw sequence data were quality checked and assembled by using SPAdes 4.0.1 using the careful correction option to reduce the number of mismatches in the final assembly with k-mer values automatically chosen by SPAdes (66). Subsequently, the assembled contigs were subjected to serovar prediction by SISTR v1.1.1 (67) and sequence typing by MLST v2.19.0 (https://github.com/tseemann/mlst). The detection of antimicrobial resistance genes (ARGs), virulence factors, and plasmids was performed by ABRicate v1.0.1 against ResFinder v4.0, VFDB, and PlasmidFinder v2.1.1, respectively (http://www.github.com/tseemann/abricate), while the scan of point mutations was conducted by Staramr software against PointFinder v1.9 (https://github.com/phac-nml/staramr). All bioinformatic analyses were performed on our in-house Galaxy platform, as documented previously (6).

Phylogenomic analysis was performed based on the core single-nucleotide polymorphisms (SNPs). All of the studied Salmonella isolates (*n* = 105) were analyzed using Snippy v4.4.4 to obtain an SNP alignment, and a phylogenetic tree was built using IQ-TREE v1.6.12 with the TVM+F model transversion model [TVM]: variable base frequencies, variable transversion rates, transition rates equal [PAUP*: abcdbe, PAML: abcdea] and 1,000 bootstraps, as previously described (40). We also coded the sequence type, geographical region, and relationship between farms with a heatmap.

### Cooccurrence analysis of plasmids and antimicrobial resistance determinants.

To *in silico* investigate antimicrobial resistance genes and their association with plasmids, ResFinder v4.0, available on our Galaxy platform, was employed for the *in silico* prediction of acquired antimicrobial resistance genes in the genomes. For a hit to be reported by ResFinder, it needs to cover at least 75% of the gene length with a sequence identity above 75%. The prediction of the location of antimicrobial resistance genes in plasmids was performed using a stepwise procedure by combining the results of resistance prediction and plasmid identification steps. The output from ResFinder gives the contigs on which antimicrobial resistance genes are located in the whole-genome assembly generated by SPAdes. Contigs identified by ResFinder were searched to determine whether they were the same as those carrying plasmid replicons identified using PlasmidFinder. Cooccurrence was established only if the detected plasmid replicon contig also contained an antimicrobial resistance determinant.

### Data analysis.

The analysis of the obtained results and the generation of figures were performed using GraphPad Prism version 8 software (GraphPad, San Diego, CA, USA), and the percent concordance between phenotypic resistance to an antimicrobial category and the presence of genetic determinants conferring antimicrobial resistance to the corresponding category was determined according to a method described previously (68).

### Ethics statement.

No ethical approval was deemed necessary for this study. Oral agreement and permission were obtained from the farmers before sampling.

### Data availability.

The data sets generated for this study can be found under NCBI BioProject accession number PRJNA828009.
